# Variations in the Physical Properties and Microbial Community of Dairy Cow Manure—Implications for Testing and Efficacy of Footbathing Products

**DOI:** 10.3390/ani13142386

**Published:** 2023-07-22

**Authors:** Maeve A. Palmer, Martin J. Garland, Linda D. Stewart, Sarah J. Helyar, Niamh E. O’Connell

**Affiliations:** 1 Institute for Global Food Security, School of Biological Sciences, Queen’s University Belfast, Belfast BT9 5DL, UK; 2 Functional Chemical Research Centre, Kersia Group UK and Ireland Ltd., Belfast BT36 4TY, UK

**Keywords:** dairy cow, digital dermatitis, microbiome, footbathing, disinfectant

## Abstract

**Simple Summary:**

Digital dermatitis is a major welfare problem for dairy cows, causing lameness and pain. It is infectious and caused by bacteria; therefore, walking cows through footbaths filled with disinfectant is a common part of control programmes. As the cows walk through the footbaths they can defecate, contaminating the disinfectant with organic matter and bacteria, which can reduce the disinfectant performance. The properties of manure (including pH, dry matter and bacterial community composition) might impact how manure contamination affects disinfectant performance. These properties might vary depending on the production system (including diet) of the animal. This study investigated the physical properties and bacterial composition of dairy cow manure from two production systems and examined whether the source of contaminating manure impacted the efficacy of footbathing disinfectants. Differences in dry matter content and bacterial community composition were found between manure samples from different production systems. When manure was added to three disinfectant products, the source of the manure affected the performance of two of the products. The source of manure used in testing products could therefore affect how well products perform. Some products might perform better on one farm than another, depending on the properties of the manure that contaminates footbaths.

**Abstract:**

Footbaths containing disinfectants are used on dairy farms to reduce the spread of digital dermatitis; however, they commonly become contaminated with manure. This trial investigated the physical properties and microbial composition of dairy cow manure from two production systems and examined whether the source of manure impacted the efficacy of footbathing disinfectants. Manure was collected from eighteen dairy cows, nine housed and fed grass silage (HOUSED) and nine at pasture (PASTURE). The pH and dry matter content was determined, total DNA was extracted and the region v3-v4 of the 16s rRNA gene sequenced. The efficacy of formalin and two trial products (TP1: peracetic acid and hydrogen peroxide; TP2: chlorocresol and triamine) was evaluated when mixed with manure from the two production systems. Production system differences were found in manure dry matter content, bacterial microbiome and the efficacy of both trial footbathing products but not formalin. The properties of manure affected the results of laboratory testing and therefore have the potential to influence footbathing disinfectant efficacy when footbaths are contaminated with manure. Further research into the impact of organic contaminants on the efficacy of disinfectants could facilitate the development of improved testing programmes and disinfectant products.

## 1. Introduction

Digital dermatitis (DD) is a widespread, infectious disease that causes ulcerative lesions on the heels of dairy cattle, leading to pain and lameness [1], and a reduction in productivity [2]. The disease is polybacterial; however, the primary etiological agents are thought to be spirochaete bacteria of the genus *Treponema* [3,4]. Disinfectant footbaths are commonly used on dairy farms as part of a DD control programme [5,6]. Factors that are considered to influence the success of footbathing programmes to control DD include the disinfectant product used and footbath design [6], the frequency of footbathing [7,8] and the number of cows passing through before the disinfectant solution is replaced [9]. The number of cows passing through can have a considerable impact because the footbaths become increasingly contaminated with manure as animals walk through and defecate—it has been estimated that after 200 cow passes the contamination level is approximately 21.2 g L^−1^ organic matter [10] or up to 20% of the volume of the footbathing product [11]. This addition of manure could reduce the efficacy of the footbathing disinfectants used, as the efficacy of many, but not all, disinfectants is reduced through the addition of organic matter [12,13] and non-target microorganisms [13].

Although there is an awareness that the volume of manure added to a footbath could reduce the efficacy of the disinfectant used, the properties of the manure might also be important in this respect. For example, the performance of some disinfectants is affected by pH [14] and disinfectants can also differ in their efficacy against different types of bacteria [13]. Changes in farming practices mean that dairy cows are currently kept under a range of production systems: some are kept in a pasture-based system for at least part of the year (with grazed grass the main forage), while others are housed or kept on dry lots year-round (with a variety of diets). Differences in the diet and production system of the animals may lead to differences in the bacterial community composition in the manure [15] and also in other manure properties, such as consistency [16], starch content [17] and pH [18].

If the efficacy of footbathing products can be affected by the properties of contaminating manure, this would have an impact not only on farms but also during laboratory research and the testing of products. Claims of bactericidal efficacy for footbathing products are currently evaluated in the European Union using standardised laboratory tests—EN 1656 [19] and EN 16437 [20]. To evaluate the impact of organic matter contamination on product efficacy these tests use sterile, standardised ‘interfering substances’, such as bovine albumin and yeast extract. This approach ensures repeatability of tests but can lead to better product efficacy results than when a more realistic substance, such as animal faeces, is used as a contaminating substrate [21,22]. To examine the impact of natural contamination when the product is in use on farms, some commercial companies and academics use dairy cow manure in the laboratory testing of candidate products [11,23]. The manure used in testing can at times be sterilised by autoclave before use, which could remove one source of variation—the bacterial community. However, whether manure that is autoclaved before use has the same impact on disinfectant efficacy as raw manure has not yet been reported.

The aims of this study were: (1) to evaluate differences in the characteristics (pH, dry matter content and bacterial microbiome) of dairy cow manure sourced from two common production systems on the same farm and (2) to determine whether manure source and sterilisation affect the efficacy of footbathing disinfectants when evaluated in a laboratory. To our knowledge, this is the first study to evaluate the effect of source of contaminating manure on the efficacy of footbathing disinfectants. The disinfectants used were chosen to represent different active ingredients used in footbaths and included formalin, a trial product containing peracetic acid (Trial Product 1, TP1) and a trial product containing chlorocresol and triamine (Trial Product 2, TP2).

## 2. Materials and Methods

### 2.1. Ethical Approval

No animals were used directly in the course of this work—there was no contact with animals during the collection of manure samples and at no time were animals in contact with any of the disinfectant products tested in the laboratory. The animals that produced the manure used in this study were selected from animals enrolled in existing trials at the Agri-Food and Biosciences Institute (AFBI), Hillsborough, Northern Ireland. All experimental procedures in those trials were conducted under an experimental license granted by the Department of Health, Social Services and Public Safety for NI in accordance with the Animals (Scientific Procedures) Act 1986 and were approved by the AFBI Hillsborough Animal Welfare Ethical Review Body.

### 2.2. Animals and Manure Collection

This study used manure from 18 dairy cows at AFBI, Hillsborough, Northern Ireland. The animals were enrolled in studies examining different production systems, which entailed different housing conditions and diets. Nine animals were part of a group that was housed in a cubicle house and fed a diet of grass silage supplemented with 10 kg concentrates per day (HOUSED). The other nine cows were part of a group that was kept at pasture (day and night) with a diet of grazed grass supplemented with 5.5–7.0 kg of concentrates per day (PASTURE). The group at pasture was made up of Holstein Friesian and Holstein Friesian × Jersey animals and the housed group contained both of these breeds plus some animals which were a cross of Holstein Friesian with Swedish Red × Jersey × Holstein Friesian. All animals were parity 1–4 and mid to late lactation (143–360 days post-partum at the time of sample collection).

Manure was collected from the animals’ environment (cubicle house or pasture) shortly after morning milking. Animals were observed until spontaneous defecation occurred, samples were immediately collected into sterile containers and animal identity was recorded. Samples were transported immediately to the laboratory and then thoroughly mixed.

### 2.3. Physical Properties of Manure

The pH of each manure sample was measured at an environmental temperature of 20 °C using a digital pH meter (Hannan Instruments, Bedfordshire, UK). The percentage dry matter content of 50 g of each manure sample was calculated after drying at 60 °C for 72 h ((dry weight/fresh weight) × 100).

### 2.4. Manure Microbiome Profiling

Aliquots of the fresh manure (50 mL) were frozen at −20 °C shortly after collection and were kept frozen until DNA extraction. After the manure was gently thawed and again thoroughly mixed, DNA was extracted using a Qiagen Powersoil Pro kit (Qiagen UK, Manchester, UK), according to the manufacturer’s instructions. The extracted DNA was stored at −20 °C until it was forwarded to the Genomics Core Technology Unit at Queen’s University Belfast for library preparation and sequencing.

The library was prepared in a two-step PCR process. The v3-v4 region of the 16s rRNA gene was amplified using primers 515F (5′-GTGCCAGCMGCCGCGGTAA-3′) and 806R (5′-GGACTACHVGGGTWTCTAAT-3′) [24] with overhang adapters using KAPA HiFi HotStart Ready Mix (Kapa Biosystems, Inc. Wilmington, MA, USA). The conditions for first stage PCR were as follows: initial denaturation at 95 °C for 3 min, followed by 25 cycles consisting of 98 °C for 30 s, 55 °C for 30 s, and 72 °C for 30 s, followed by a final extension step at 72 °C for 5 min. The PCR products were cleaned using KAPA Pure Beads (Roche, Wilmington, MA, USA), and the size of PCR products were identified on a Fragment Analyzer (Advanced Analytical Technologies Inc., Ames, IA, USA). A second stage (index) PCR step was then carried out to attach the multiplexing indices and Illumina sequencing adapters, using the Nextera XT Index kit (Illumina, San Diego, CA, USA). PCR was again performed using KAPA HiFi HotStart Ready Mix (Kapa Biosystems), with an initial denaturation at 95 °C for 3 min, then eight amplification cycles: 95 °C for 30 s, 55 °C for 30 s and 72 °C for 30 s, followed by a final extension at 72 °C for 5 min. The products were cleaned using KAPA Pure Beads, and product size was determined using the Fragment Analyzer.

DNA concentration was measured using a Qubit Fluorometer 3.0 (Invitrogen, Carlsbad, CA, USA) and adjusted to 4 nM. DNA was sequenced using an Illumina Miseq (Illumina, San Diego, CA, USA) with 250 bp paired end reads. The generated 16S rRNA raw gene sequences were pre-processed to remove any remaining barcodes and adapters using Trimmomatic v0.3831 [25] and initial quality control was carried out using FASTQC (Babraham Institute; https://www.bioinformatics.babraham.ac.uk/projects/fastqc (accessed on 03 August 2020)).

Sequences were passed through the Dada2 v3.11 [26] pipeline in R v4.0 [27] for further quality control and trimming, the merging of the forward and reverse reads, chimera removal and the production of an amplicon sequence variant (ASV) table. Taxonomic assignment was carried out with a Bayesian classifier and the SILVA v138 training set. A negative extraction control was processed and sequenced alongside the samples. The results for the negative control sample were then used with the Decontam v1.1 [28] package in R to identify any potential contaminating sequences in the study data. Sequences were processed using the Phyloseq package v1.32.0 [29] in R (v4.0) to produce alpha (within sample) and beta (between samples) diversity indices and plots (NDMS plots and clustering dendrogram). To account for variation in the total number of reads in each sample, count data were transformed using a variance stabilising transformation prior to the calculation of beta diversity indices [30].

The raw FASTA files of the sequence data were submitted to European Nucleotide Archive and are accessible at the following link: http://www.ebi.ac.uk/ena/data/view/PRJEB36393 (accessed on 15 April 2022).

### 2.5. Laboratory Testing of Disinfectant Efficacy When Contaminated with Manure

A subset of the fresh manure samples was evaluated via quantitative suspension tests. Ten manure samples were selected—five from the HOUSED and five from the PASTURE groups. Samples from each group were selected to provide a similar mean cow parity between groups and a range of manure textures (as judged by visual inspection) within each group. Care was taken to select a subset that represented the range of manure textures in each group.

To determine the initial aerobic bacterial load in each manure sample before the samples were used in testing, the number of colony-forming units (CFU) of aerobic bacteria per ml present in the fresh manure was determined by serial dilution of the manure samples, followed by plating in duplicate on tryptone soy agar (Oxoid, Thermo Fisher Scientific, Loughborough, UK). Plates were incubated for 24 h at 37 ± 1 °C and the number of colonies counted.

Trial product 1 (TP1) contained active ingredients in the following ranges: acetic acid (10–25%), peracetic acid (5–10%) and hydrogen peroxide (8–35%). Trial product 2 (TP2) contained Lactic acid (10–30%), benzenesulfonic acid (1–10%), 4-chloro-3-methylphenol (1–10%) and 2-(2-butoxyethoxy)ethanol (1–10%). The formalin solution contained formaldehyde (30–50%) and ethanol (3–10%). Quantitative suspension tests were carried out to test the efficacy of the three footbathing products when altered to contain 20% raw manure (*v*/*v*) from each of the 10 animals, using a protocol modified from BS EN 1656:2009 [31]. The three disinfectant products were tested with each of the ten manure samples, a total of 30 quantitative suspension tests.

Disinfectants were tested at a final concentration of 5% (*v*/*v*) after dilution with tap water. *Staphylococcus epidermidis* (from existing laboratory stocks) was used as the challenge bacteria, as a representative of the Gram-positive bacteria commonly found on the skin of dairy cows [32,33]. Contact time with the disinfectant was 30 s at a temperature of 20 °C (the short contact time was chosen to reflect the short contact time when products are used in footbaths on farms). The neutraliser used was 900 mL typtone soya broth (Oxoid, Thermo Fisher Scientific, Loughborough, UK) with 100 mL Tween-80, 5 g sodium thiosulphate (Sigma-Aldrich, Dorset, UK), 30 g lecithin, 1 g L-histidine and 1 g L-cystine (Alfa Aestar, Heysham, UK).

A 1 mL aliquot of a suspension of *S. epidermidis* in tryptone salt diluent (Bio-Rad, Hertfordshire, UK) was added to 9 mL of disinfectant–manure mixture to achieve a final manure concentration of 20% and a final disinfectant concentration of 5%. This was well mixed and left for 30 s. After mixing again, a 1 mL volume of this mixture was added to 9 mL of neutraliser solution to halt the action of the disinfectant. After a neutralisation time of 5 min, the neutralised mixture was mixed again, a 100 µL aliquot was plated onto tryptone soy agar in duplicate and the plates were incubated for 24 h at 37 ± 1 °C. The average number of CFU present in the suspension after disinfectant contact and neutralisation was determined by counting the number of colonies on each plate and working out the average CFU/mL value for the duplicate plates.

To calculate the log_10_ reduction value for the test, the BS EN 1656:2009 [31] standard process was followed. Firstly, the log_10_ of the post-disinfectant CFU/mL was calculated. The number of CFU in the *S. epidermidis* suspension before disinfectant contact was determined and the total number of aerobic bacteria in the sample of raw manure (determined as described above) was added to this value to give a value for the total bacterial challenge before the start of the disinfectant contact. The log_10_ of the initial bacterial challenge was then calculated, and the log_10_ of the number of bacteria after disinfectant contact was subtracted from this to yield the log_10_ reduction value for each manure–disinfectant combination (with a higher log_10_ reduction value demonstrating a better product efficacy). This was repeated for each of the manure–disinfectant combinations (30 tests in total). Experimental condition controls, neutraliser toxicity controls and dilution–neutralisation validations were carried out for each disinfectant, as outlined in BS EN 1656:2009 [31].

To investigate whether manure that had been sterilised had the same impact on the disinfectant as raw manure, quantitative suspension tests (outlined above) were used to test the efficacy of one of the products (TP2 disinfectant) and manure samples from the same animals after the manure had been sterilised by autoclaving (121 °C for 15 min).

### 2.6. Statistical Analysis

Comparisons between the pH and dry matter content of manure, the number of reads from the HOUSED and the PASTURE samples, alpha diversity parameters and the number of post-partum days of animals that provided the samples were carried out using *T* tests for independent samples in SPSS v26 (IBM, New York, NY, USA), and Levene’s test used to check for equality of variances. The parity of animals in the two groups was compared using Fisher’s exact test in SPSS v26. Data for the log_10_ reduction in bacteria caused by the three footbathing products were not normally distributed so comparisons were carried out using Friedman’s related sample two-way ANOVA by rank and post hoc tests, using Bonferroni’s correction to identify where differences lay. Comparisons of the log_10_ reduction in bacteria when the same product was tested with raw or sterile manure was tested using the Wilcoxon’s related sample signed rank test.

Differences in the overall composition of the bacterial microbiome were analysed using a permutational multivariate ANOVA using Adonis in R v4.0 [27]. Differences in the percentage composition of different phyla between samples from the HOUSED and the PASTURE animals were analysed using Mann–Whitney U-tests, with Bonferroni’s correction applied. For all of the above analyses, results were considered significant when *p* < 0.05. The DESeq2 package v1.24.0 [34] was used to identify amplicon sequence variants (ASVs) with a significantly different occurrence between the HOUSED and the PASTURE groups. For this analysis, results were filtered to include only ASVs that had a base mean occurrence of >10 reads per sample, the Benjamini–Hochberg adjustment for multiple tests was applied and an adjusted *p*-value of <0.01 was considered significant.

## 3. Results

### 3.1. Group Comparison and Physical Properties of Raw Manure

The physical characteristics and within-sample microbiome diversity metrics of the manure samples are listed in Table 1. The manure samples from housed cows had a higher mean dry matter content than those of the cows at pasture (*p* < 0.01), but there was no difference in the mean pH of the manure samples from the animals in the two groups (*p* = 0.291; Table 1). When the entire group of 18 animals was considered, the parity of animals in the PASTURE group was lower than that of animals in the HOUSED group (mean ± SE PASTURE = 1.56 ± 0.18, HOUSED = 3.22 ± 0.28; *p* < 0.01). There was no difference in the mean number of days post-partum between the animals in the two groups (mean ± SE PASTURE = 209.7 ± 6.63, HOUSED = 205.2 ± 23.2; *p* = 0.858).

### 3.2. Overall Profile of Manure Microbiome in Housed and Pasture Cows

A total of 785,318 sequencing reads passed the quality control filters for the 18 samples, with the minimum number of reads per sample at 37,380. The negative control sample had only 13 sequencing reads and the Decontam (v1.1) package did not identify any contaminating sequences. No difference was found in the mean number of quality filtered sequencing reads or in Shannon diversity (a measure of richness and evenness of the bacterial community) between the manure samples from the HOUSED and PASTURE animals (Table 1). The species richness (number of ASVs identified) of the PASTURE manure samples was significantly greater than the species richness of HOUSED manure samples.

**Figure 1:**
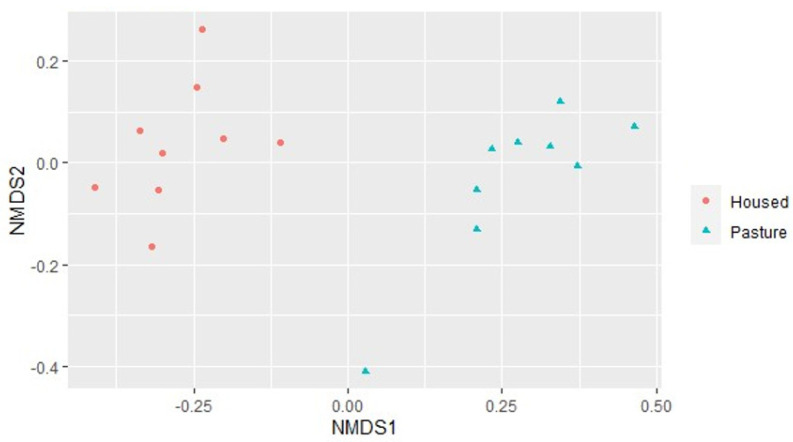
NDMS ordination plot of Bray–Curtis distances between VST transformed count data, showing overall bacterial community composition in manure samples. Samples with a more similar community composition are closer together. Samples from HOUSED (fed silage plus concentrates) shown with red circles (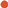), samples from PASTURE (fed grass plus concentrates) shown with blue triangles (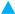).

**Figure 2:**
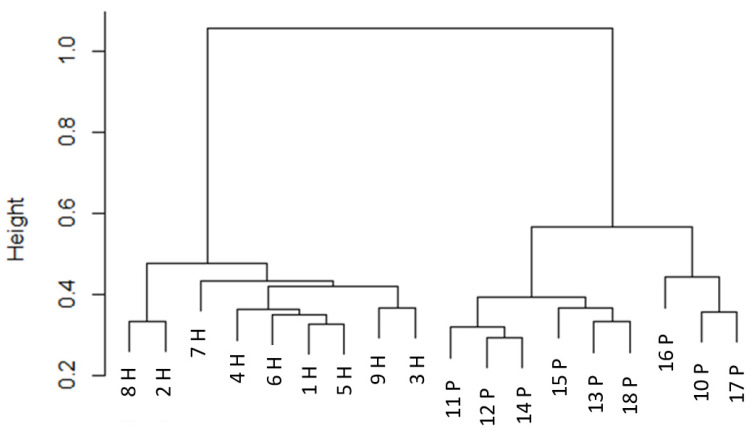
Clustering dendrogram (produced using Ward D2 method with Bray–Curtis distances of VST transformed data) showing the grouping of the samples from pastured cows and housed cows on the basis of bacterial community composition. Branches are named with the ID of the cow the sample came from—samples with an “H” from housed cows and with a “P” from pasture cows.

Figure 1 shows the diversity of samples from the nine pastured and nine housed cows, demonstrating a separation between the samples from the HOUSED and the PASTURE animals and variation within groups. A clustering dendrogram (Figure 2) similarly shows that samples from the HOUSED and the PASTURE groups cluster together and are therefore more similar to one another than to samples from the other group. This difference in the bacterial community composition was confirmed via permutational ANOVA analysis, which showed a significant difference between the composition of manure bacterial microbiomes from HOUSED and PASTURE animals (*p* < 0.01).

The median percentage composition of the microbiome of housed and pasture cows according to the bacteria and archaea of different phyla and the significance of the differences between groups (adjusted for multiple comparisons) is shown in Table 2. Significant differences were found in the proportions of many Gram-positive and Gram-negative phyla, as well as Archaea (Table 2). The largest numerical differences were in the proportion of *Firmicutes* (higher in pasture cow manure), *Euryarchaeota* (higher in housed cow manure) and *Bacteroidota* (numerically higher in housed cow manure, but the difference was not statistically significant).

A differential expression analysis (including only ASVs that had a base mean abundance of greater than 10) found 103 ASVs that were at significantly different levels in the two groups. The top 20 most abundant of those that differed between groups included both bacteria and archaea. The top 20 genera that contained representatives at a higher abundance in the group at pasture included *Paeniclostridium* and *Romboutsia*, *Alistipes*, *Fibrobacter* and *Catenibacterium*. At a higher abundance in the housed group were ASVs from the bacterial genera *Bacteroides*, *Prevotellaceae*, *Succinivibrio*, *Lachnospiraceae* (NK3A20 group) and *Blautia* and the archaeal genera *Methanobrevibacter* and *Methanosphaera*.

### 3.3. Efficacy of Footbathing Products When Mixed with Raw Manure

**Figure 3:**
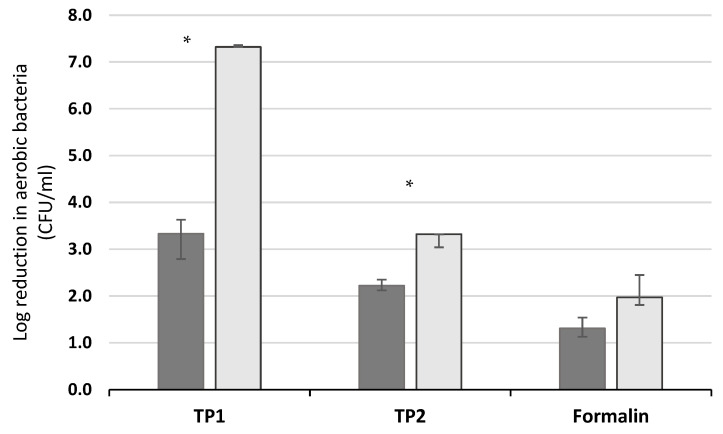
Median log_10_ reduction in bacteria numbers caused by disinfectants containing manure from dairy cows housed (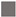) and at pasture (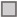) when tested using *S. epidermidis* (error bars show interquartile range). Significant differences between the log_10_ reduction values when using manure from housed and pastured animals are marked with an *.

There was no difference in the (log_10_ transformed) initial number of CFU/mL of aerobic bacteria between manure from animals in the HOUSED (median = 6.00, interquartile range (IQR) = 0.60) and PASTURE (median = 5.70, IQR = 1.54; *p* = 0.841) groups before samples were used in quantitative suspension tests. The results of the quantitative suspension tests using 20% raw manure as an interfering substance are shown in Figure 3. Both Trial Products 1 and 2 showed higher median log_10_ reductions in bacterial numbers when manure from the PASTURE group was used in testing than when manure from the HOUSED group was used (both *p* < 0.01), but no such difference was seen for formalin (Figure 3).

The efficacy of one of the products (TP2) was also determined using manure that had been sterilised via autoclaving before it was added to the disinfectant. The median log_10_ reduction in bacterial numbers obtained by TP2 was greater when manure was sterilised by autoclaving before testing (median log_10_ reduction with sterile manure = 3.88, IQR = 1.92; median log_10_ reduction with raw manure = 2.49, IQR = 1.13; *p* < 0.01), indicating that the disinfectant was more effective when mixed with sterile manure than when mixed with raw manure. When sterilised manure was used in testing no difference was found between the efficacy of the disinfectant with manure from the HOUSED or PASTURE groups (log_10_ reduction in bacteria: HOUSED = 4.76, IQR = 2.00; PASTURE = 3.73, IQR = 1.91; *p* = 0.548).

## 4. Discussion

A wide range of disinfectant products are available with differing active ingredients and modes of action. To ensure that the best products are selected for particular uses, we must understand the interactions between disinfectants and the environment in which they are expected to work, particularly any substances that may interfere with the efficacy of the product when in use. The current standards used for testing the efficacy of disinfectant products vary geographically, with each region specifying required tests and standards (summarized by [35]). The EU standards for the testing of footbathing disinfectants are EN1656 [19] and EN16437 [20]. The tests that comprise these standards do not evaluate the impact of the presence of manure during the testing process, but instead use proxy interfering substances (including yeast and bovine serum albumin) to determine how the activity of the disinfectant might be affected by contaminating organic substances. The major advantage of this approach is that it allows consistency and reproducibility between laboratories and over time [35]. It is also important, however, to evaluate how products will perform on farms when challenged with manure contamination, as this is an inevitable part of the dairy cow footbathing process when using common footbath designs. A small number of published studies have used dairy cow manure as an interfering substance when evaluating the performance of footbathing products [11] or candidate active ingredients [23]. These studies used fresh manure or autoclaved manure and reported the volume added but not the characteristics or source of the manure and whether manure was from one or more animals.

The first aim of this study was to evaluate some of the physical characteristics of manure samples taken from two groups of animals, HOUSED and PASTURE. One of the physical characteristics measured was pH, as it is known that the pH of a solution has an impact on the efficacy of some disinfectants [14]. Although no significant difference in manure pH was detected between samples from animals in the two groups in the current study, there was variation in pH with a range between pH 6.43 and 7.15. Evidence from the literature suggests that variation in pH between dairy cows on different diets can occur; for example, Ferreira et al. [18] found that both calcium and protein content of the diet altered the manure pH in Holstein heifers, and Ireland-Perry et al. [36] report that a diet higher in fibre was associated with manure that had a lower pH. More recently, Lee et al. [37] found a tendency towards reduced manure pH when soybean meal was replaced with dried distillers’ grains in the diet of dairy cows, and Khorrami et al. found that manipulating dietary starch also affected faecal pH [38]. As the pH of the manure is likely to be confounded with, for example, microbiome [39] and protein levels [18], it would be difficult to study the relationship between manure pH and disinfectant efficacy in isolation, but an appreciation that the manure pH can vary between animals and diets and that this could impact the efficacy of some types of disinfectant is warranted, and recording the pH of any manure used in laboratory testing would help reproducibility.

The second physical property of the manure tested in the current study was the dry matter content. The manure samples from the group of cows at pasture were found to have a lower dry matter content than the manure of the housed group. This is not unexpected, as differences in the dry matter content (or consistency) of manure have been found previously when dairy cows were fed different diets [16,36]. Differences in the dry matter content of manure samples used in laboratory testing could cause difficulties in terms of reproducibility, as the amount of manure added is generally measured by volume [21,23], and if dry matter content varied, then the same volume of manure would contain a different amount of organic matter.

In addition to the pH and dry matter content of the manure, the composition of the bacterial and archaeal microbiome of each manure sample was also determined, as manure added to the footbathing disinfectant (either in the laboratory or on farm) adds a bacterial load as well as organic matter. There was no difference in the initial aerobic bacterial load of the manure samples, but manure from the two groups of animals showed differences in both richness and community composition. The two groups in this study differed in terms of current diet and immediate environment (and animals in the group at pasture had a lower mean parity) but were reared together and situated within the same farm complex. No direct comparison with this situation could be found in the literature; however, previous studies have found differences in the microbiome due to both diet and production system. An extensive survey of the faecal microbiome of Californian dairy farms by Hagey et al. [15] examined diversity by both housing type and diet and found several aspects of the diet that were associated with differences in species richness, including forage and concentrate type. In beef cattle, Shanks et al. [17] found that the richness, diversity and composition of the manure microbiome were all affected by the type of diet that the animals were fed.

An examination of the bacterial and archaeal community composition identified a significant difference between the two sets of samples, as well as differences in the proportions of particular phyla and genera in the manure of the two groups of cows. The two phyla found in the highest proportions in the current study were *Firmicutes*, which was higher in manure from pasture cows (58% vs. 52% in housed), and *Bacteroidota* (no significant difference between groups, 36% in pasture and 39% in housed cows). *Firmicutes* and *Bacteroidota* were also identified as the phyla with the highest abundance in dairy cow manure samples in other recent studies [15,40,41]. Although they are commonly the two most abundant, the proportions of these phyla vary widely between and within studies; for example, Li et al. [39] found that in Holstein dairy cows fed a total mixed ration diet the proportion of the faecal microbiome made up of *Firmicutes* was 70% in spring and 87% in summer, while *Bacteroidota* was found to contribute 25% in spring and 4% in summer.

Differences in the composition of the bacterial microbiome could have consequences for the efficacy of disinfectant products, which come into contact with the manure samples. Gram-negative bacteria (including those of the phylum *Bacteroidota*) have an outer membrane that acts as a barrier, limiting the entry of many antimicrobial agents [14]. This means that Gram-negative bacteria are generally less susceptible to disinfectants than the majority of Gram-positive bacteria, including members of the phylum *Firmicutes* [14].

There were also differences found in the proportion of two archaeal phyla between the two sets of manure samples. Although Archaea have now been detected in many different environments and there is growing understanding of their importance in ecosystems [42], there is still a comparative lack of knowledge about this group, and studies of disinfectant efficacy against representatives of the Archaea are not available. The archaeal genera which were found to be represented at a higher proportion in the manure samples from housed animals (i.e., *Methanobrevibacter* and *Methanosphaera*, are both methanogens that have been previously reported as components of the rumen microbiome [43,44].

Variation in manure microbiome composition adds an element of variability to laboratory testing carried out with fresh manure, and this has the potential to negatively impact reproducibility. To remove the impact that variation in the microbiome might have on the results of product testing, one option would be to sterilise the manure using an autoclave before using it for testing. This was the approach used by Hartshorn et al. [23] and it also has the advantage that the manure can then be aliquoted and frozen so that all testing does not need to take place at the same time and within hours of manure collection. There is, however, a potential disadvantage to this practice—in the current study, the results observed when using sterilised manure did not follow the same pattern as those when using fresh manure; therefore, the results produced might not accurately reproduce what is actually occurring on farms.

Before this study was carried out, both the trial products (TP1 and TP2) had been assessed for efficacy, according to the EN 1656:2009 standard [31], and had met the required standard (a minimum of a 5 log_10_ reduction in bacteria CFU, equivalent to a 99.999% reduction in the number of colony-forming units present) when tested using the standard interfering substances (bovine albumin plus yeast extract). Under the conditions used in the current study, with 20% of the disinfectant volume replaced by manure, the log_10_ reduction in bacterial numbers was found to be less than 5 for all but one of the disinfectant–manure combinations (Trial Product 1 plus manure from animals at pasture). These findings of a reduction in efficacy are in line with those of McLaren et al. [21] and Gosling et al. [22], who found a lower disinfectant efficacy against *Salmonella* in a testing model that included laying hen, turkey or duck faeces compared to when using the standardized, sterile interfering substances.

During the current study, in addition to the fact that all but one of the product-manure combinations gave a lower log_10_ reduction in bacterial numbers when manure was used in testing, the group of animals that the manure came from had a significant impact on the resulting product efficacy. The difference in efficacy between the addition of manure from the HOUSED or PASTURE group was greatest for Trial Product 1 (containing peracetic acid) and was also significant for Trial Product 2 (containing chlorocresol). The three products assessed have different active ingredients, and it is possible that the interaction between the manure characteristics (including the bacterial microbiome) and the mode of action of the active ingredients in the disinfectant products is the cause of this difference in response by the products. The differing impact of manure source, depending on the disinfectant product tested, adds to the complex picture in which many physical characteristics of manure interact. The current study was designed in order to determine whether differences in manure properties might be found between groups of similar animals on differing diets, and whether manure from these groups might have a different impact on the efficacy of disinfectant products. This design does not allow the relationships between individual manure characteristics and product efficacy to be extracted, in part because a number of the factors measured are not only affected directly by diet but are also influenced by each other (for example manure pH influences the microbiome [39]).

Further work on dairy farms, taking samples from footbaths while they are in use, would be necessary to confirm the impact of manure contamination on product efficacy on farms, and whether this is affected by the characteristics of the manure. To determine whether this translates into differences in digital dermatitis control, concurrent monitoring of the prevalence of digital dermatitis lesions would be required. The extension of the current laboratory work using a higher number of species of test bacteria, including *Treponema* spp. that are thought to be the causative agents of digital dermatitis [3], would provide very useful additional information.

As the farming industry deals with requirements to reduce the use of antibiotics in food production, effective cleaning and disinfection is of growing importance to maintain the health, welfare and productivity of farm animals. Considering the damaging impacts that many of the chemicals used in cleaning and disinfection products can have on human and animal health and on the environment, it is important that these substances are used in the most efficient and effective way possible. Due to advances in genetic sequencing technology, it is now possible to explore the composition and diversity of the bacterial microbiomes present in different environments on farms and in the organic matter (including manure) that often contaminates disinfectant products when used as footbaths. Further research will continue to examine the impact that the microbiome of contaminating organic substances might have on the efficacy of cleaning and disinfection regimens.

## 5. Conclusions

This study found that dairy cow manure from two production systems on one farm differed in physical properties and bacterial community composition. When this manure was used to replicate high contamination levels as part of a laboratory footbathing product testing programme, the source of the manure samples was associated with a difference in the efficacy of two of the three disinfectant products. These findings suggest that if manure is used as part of the testing of footbathing disinfectants, the properties of the manure could affect the test results. Further research is required to determine whether the properties of contaminating manure might also affect the performance of footbathing disinfectants when they are used on farms.

## Figures and Tables

**Table 1:** Physical characteristics (mean pH and dry matter content) and mean bacterial richness and diversity measures of manure samples (±SE) taken from animals in the HOUSED and PASTURE groups.

	HOUSED	PASTURE	Statistical Significance
Number of samples	9	9	
Manure pH	6.63 ± 0.09	6.76 ± 0.07	*p* = 0.291
Manure dry matter content (%)	12.80 ± 0.61	10.40 ± 0.52	*p* < 0.01
Number of sequencing reads	44,229 ± 609	43,032 ± 915	*p* = 0.293
Species richness	706.78 ± 26.76	857.22 ± 31.05	*p* < 0.01
Shannon diversity	5.54 ± 0.06	5.72 ± 0.08	*p* = 0.093

**Table 2:** Summary of median relative abundance of phyla from samples from pasture and housed cows and the statistical significances of the differences between groups. Significance values are adjusted for multiple comparisons.

Phylum	Median Relative Abundance (%)		Adjusted *p*-Value
	HOUSED	PASTURE	
Bacteria			
* Firmicutes*	51.64	58.40	<0.05
* Bacteroidota*	39.25	36.03	1.000
* Spirochaetota*	0.81	1.45	1.000
* Proteobacteria*	1.09	0.45	0.882
* Fibrobacterota*	0.00	0.61	<0.05
* Actinobacteriota*	1.27	0.36	<0.05
* Patescibacteria*	0.20	0.06	<0.05
* Planctomycetota*	0.00	0.14	0.154
* Verrucomicrobiota*	0.05	0.57	0.084
* Cyanobacteria*	0.03	0.47	<0.05
* Elusimicrobiota*	0.00	0.07	<0.05
* Desulfobacterota*	0.03	0.03	1.000
Archaea			
* Euryarchaeota*	4.52	0.56	<0.05
* Halobacterota*	0.14	0.16	1.000

## Data Availability

The raw FASTA files of the sequence data were submitted to the European Nucleotide Archive and are accessible at the following link: http://www.ebi.ac.uk/ena/data/view/PRJEB36393 (accessed on 15 April 2022). The data presented in this study are publicly available at www.pure.qub.ac.uk/en/persons/maeve-palmer/datasets/ (accessed on 30 May 2023); DOI: https://doi.org/10.17034/50fdf8c9-788e-40b0-92a1-3e38effa98ab (accessed on 30 May 2023).
